# A framework of Microbial Genomic Database for clinical metagenomic pathogen diagnosis: development and multi-cohort evaluation

**DOI:** 10.3389/fcimb.2026.1938149

**Published:** 2026-09-16

**Authors:** Han Xia, Yanhua Wen, Xuming Li, Long Hu, Yaqi Yuan, Juanjuan Tian, Song Li, Yao Zhan, Xiaofei Dang, Yuting Lin, Lili Li, Yingjie Chen, Ye Zhang, Yuanlin Guan, Jun Wang

**Affiliations:** 1 Medical Research and Laboratory Diagnostic Center, Central Hospital Affiliated to Shandong First Medical University, Jinan, Shandong, China; 2 Department of Scientific Affairs, Hugobiotech Co. Ltd., Beijing, China; 3 School of Automation Science and Engineering, Faculty of Electronic and Information Engineering, Xian Jiaotong University, Xi’an, Shaanxi, China; 4 School of Basic Medical Sciences, Shandong University, Jinan, Shandong, China

**Keywords:** host depletion, microbial reference database, mNGS, pathogen detection, sequence alignment

## Abstract

**Introduction:**

Clinical metagenomic next-generation sequencing (mNGS) enables broad, untargeted pathogen detection, but its analytical performance depends on host depletion strategy, reference database composition, and alignment methodology. We developed the Clinical Microbial Genomic Database (CMGD), a clinically focused reference resource prioritizing medically relevant taxa.

**Methods:**

CMGD was manually curated, clinically stratified, and included more than 18,000 microbial species. We evaluated host-depletion references, alignment and classification strategies, six published clinical cohorts, and 30 retrospective mNGS-positive clinical samples.

**Results:**

The combined GRCh38-T2T reference achieved the highest human-read depletion rate while minimizing microbial-read loss. CMGD provided broader target-species coverage than the standard Kraken2 database, and BWA-CMGD showed lower erroneous assignment rates overall, although Kraken2 yielded higher unique species-level assignment rates for many shared taxa. Across six published clinical cohorts, CMGD achieved 91.0% detection concordance with BLAST-NT and a strong read-count correlation (R^2^ = 0.97). In 30 retrospective samples, CMGD and NT showed strong correlations for total mapped reads (R^2^ = 0.99) and uniquely mapped reads (R^2^ = 0.89), with concordance correlation coefficients of 0.99 and 0.92, respectively. High sequence-mapping accuracy did not ensure reliable species-level discrimination for highly homologous taxa such as *Escherichia coli* and *Shigella flexneri*.

**Discussion:**

Clinically stratified database curation improves the analytical performance, computational efficiency, and interpretability of mNGS-based pathogen detection. Species-complex-level reporting may be more appropriate when species-level discriminatory evidence is insufficient. Prospective multicenter validation is required to establish clinical diagnostic utility.

## Introduction

Metagenomic next-generation sequencing (mNGS), which eliminates the need for microbial culture and prior knowledge of the causative pathogen, enables the simultaneous detection of bacteria, fungi, viruses, and parasites from clinical specimens in a single assay, and has become a widely adopted tool for clinical etiological identification (Luan et al., 2021). Compared with conventional microbiological methods, mNGS has demonstrated substantially superior sensitivity in pathogen detection (Liu et al., 2023; Benoit et al., 2024; Ye et al., 2024). Leveraging its rapid turnaround and unbiased broad-coverage capability, mNGS is increasingly applied across a broad spectrum of clinical scenarios, including severe pneumonia (Yang et al., 2022), central nervous system (CNS) infections (Feng et al., 2025), and bloodstream infections (BSI) in intensive care unit (ICU) patients (Liu et al., 2023). This is particularly important for critically ill and immunocompromised individuals, in whom timely and accurate pathogen detection is pivotal for guiding targeted antimicrobial therapy.

Pathogen detection via mNGS comprises two interdependent components: wet-lab experimental procedures and dry-lab bioinformatic analysis. The reliability of diagnostic outcomes depends not only on a complete bioinformatics workflow, which converts raw FASTQ sequencing data into interpretable microbial signals through sequential steps including quality control, host read subtraction, sequence alignment, and taxonomic classification, but also on the quality and composition of underlying reference databases. Host subtraction is critical to this pipeline, as clinical specimens are frequently dominated by human-derived sequences. For common clinical specimen types, including respiratory samples, blood, and body fluids such as CSF, host reads typically account for over 90% of total sequencing data and can even reach 99% (Cheng et al., 2023; Kim et al., 2024; Diao et al., 2025). Excessive host reads dilute effective microbial sequencing depth, impede the detection of low-abundance pathogens, and skew downstream analysis of microbial community composition. To address this issue, routine clinical mNGS workflows first align raw sequencing data to the human reference genome to filter out host sequences. The remaining non-host reads are then annotated against microbial databases. However, host-subtraction performance varies across tools and depends strongly on the completeness and accuracy of the host reference genome (Gao et al., 2025). Accordingly, host subtraction should be evaluated together with microbial database composition and aligner choice because these steps jointly determine which reads are retained, which taxa are searchable, and how final pathogen calls are interpreted (Gao et al., 2025).

After host subtraction, the remaining non-host reads are aligned against clinical microbial genome databases for pathogen identification. The diagnostic efficacy of mNGS is fundamentally constrained by the composition and completeness of reference databases, which effectively define the upper bound of detectable taxa (Smith et al., 2022; Martí et al., 2025). Because pathogens absent from the database produce no detectable signal and generate no explicit warning for clinicians, incomplete reference coverage inherently leads to silent false negative results (Jing et al., 2021; Smith et al., 2022). Conversely, the absence of representative genomes causes sequences to be spuriously assigned to phylogenetically related but taxonomically distinct references, producing false positive identifications through misclassification (Jing et al., 2021; Chorlton, 2024; Martí et al., 2025; Cao et al., 2026). This problem is further compounded by well-established quality issues in widely used public repositories such as GenBank, RefSeq, and the NCBI nucleotide (NT) database, on which most clinical laboratories currently depend (Diao et al., 2023; Diao et al., 2025), including taxonomic mislabeling, sequence contamination, and inappropriate inclusion or exclusion criteria (Chorlton, 2024). Yet expanding database coverage indiscriminately is not a viable solution, as the exponential growth of reference genome databases places increasingly large computational demands on taxonomic classification that are difficult to meet in time-sensitive clinical settings (Tausch et al., 2022; Tan et al., 2024; Ulrich and Renard, 2024). Therefore, the strategic curation of reference sequences that balances taxonomic breadth, sequence integrity, and computational efficiency is essential for reliable and timely mNGS-based diagnosis. Yet how to achieve this balance remains an open question, as current database design strategies exhibit fundamental trade-offs between taxonomic breadth and clinical specificity.

While type strain-focused databases offer advantages in uniformity and computational tractability, their limited scope renders them insufficient for the full spectrum of clinical diagnostic needs (Robeson et al., 2021). More broadly, most current clinical reference databases are constructed to maximize global taxonomic coverage rather than clinical utility (Dulanto Chiang and Dekker, 2020; Elbehiry and Abalkhail, 2025): genomic sequences are incorporated without clinical stratification, assigning equal representation to both clinically significant pathogens and environmentally abundant, non-pathogenic organisms (Qian et al., 2023; Que et al., 2025; Xie et al., 2025). This non-stratified architecture produces two compounding diagnostic liabilities. On one hand, clinically relevant pathogens, particularly rare or emerging ones, remain underrepresented, increasing the risk of false-negative identifications. On the other hand, the inclusion of extensive environmental sequences introduces redundancy that inflates database volume, elevates computational demands, and compromises diagnostic timeliness (Govender and Eyre, 2022). Therefore, a stratified clinical database design, encompassing pathogen inclusion, sequence exclusion, and erroneous sequence filtering must be evaluated in concert with alignment tool selection to optimize diagnostic performance (Chorlton, 2024).

The choice of bioinformatic alignment tool is another key factor influencing diagnostic performance (Simon et al., 2019; Sun et al., 2022). mNGS-based taxonomic classification can be achieved through several computational strategies, including alignment (to all sequences or taxonomically unique markers), composition (*k*-mer analysis), phylogenetics (using models of sequence evolution), assembly, or a combination of these methods (McIntyre et al., 2017). Given the dual requirements for high diagnostic accuracy and rapid turnaround time in clinical mNGS, most clinical laboratories currently use DNA-based alignment tools such as BWA (Li, 2013), Kraken2 (Wood et al., 2019), and BLAST (McGinnis and Madden, 2004) along with protein-based alignment tools like Diamond (Han et al., 2020, 2022; Diao et al., 2023, 2025). Among these approaches, *k*-mer-based classifiers such as Kraken2 enable rapid, high-throughput taxonomic assignment, but their reliance on fixed-length *k*-mer matching makes them prone to misclassification when reference coverage is incomplete. In contrast, BLAST is a widely used high-sensitivity sequence-alignment comparator, but due to its slower alignment speed, it is often used for mNGS validation (Naccache et al., 2014; Bazinet et al., 2018; Piantadosi et al., 2021). BWA, a local alignment-based approach with good alignment accuracy and relatively fast speed, has become the most widely adopted alignment tool across diagnostic platforms (Han et al., 2022; Diao et al., 2025). Importantly, the performance of these tools is intrinsically linked to the underlying reference database, and database design may affect different methods in distinct ways. Therefore, a systematic framework that jointly optimizes reference database design and alignment tool selection is essential to advance both the accuracy and efficiency of clinical mNGS diagnostics.

To address these challenges, we developed and systematically evaluated a clinical mNGS analytical framework based on the Clinical Microbial Genomic Database (CMGD), a curated and clinically focused reference resource. CMGD incorporates a tiered stratification strategy that prioritizes pathogens according to their clinical significance and representation in recognized medical databases. We systematically evaluated the effects of host subtraction strategies and alignment tool selection on CMGD analytical performance using both simulated datasets and clinical cohorts derived from in-house collections and publicly available clinical datasets retrieved from the literature. Our findings provide evidence-based guidance for optimizing both reference database design and bioinformatic pipelines, providing a basis for further evaluation of clinical mNGS workflows.

## Materials and methods

### Data collection and CMGD database construction

Microbial taxa lists were collected from multiple public repositories containing WHO priority pathogen list, Karius pathogen list (https://kariusdx.com/our-solution/pathogens?product=spectrum), gcPathogen, FDA-ARGOS, FungiDB, WormBase ParaSite, RefSeq and GenBank (Howe et al., 2017; Basenko et al., 2018; Tacconelli et al., 2018; Blauwkamp et al., 2019; Sichtig et al., 2019; Guo et al., 2024). Genomic sequences were subsequently retrieved from public databases, including GenBank (Release 260), RefSeq (Release 224) (O’Leary et al., 2016), FDA-ARGOS (V-1.64), gcPathogen (202404), FungiDB (Release 68), WormBase ParaSite (WBPS18), and RVDB (v28.0) (Goodacre et al., 2018) (**Figure 1A**) and an in-house dataset of *de novo* assembled genomes of clinical isolates cultured from patient specimens. Comprehensive metadata, including sample information, sequencing parameters, and assembly quality metrics, was systematically documented.

**Figure 1:**
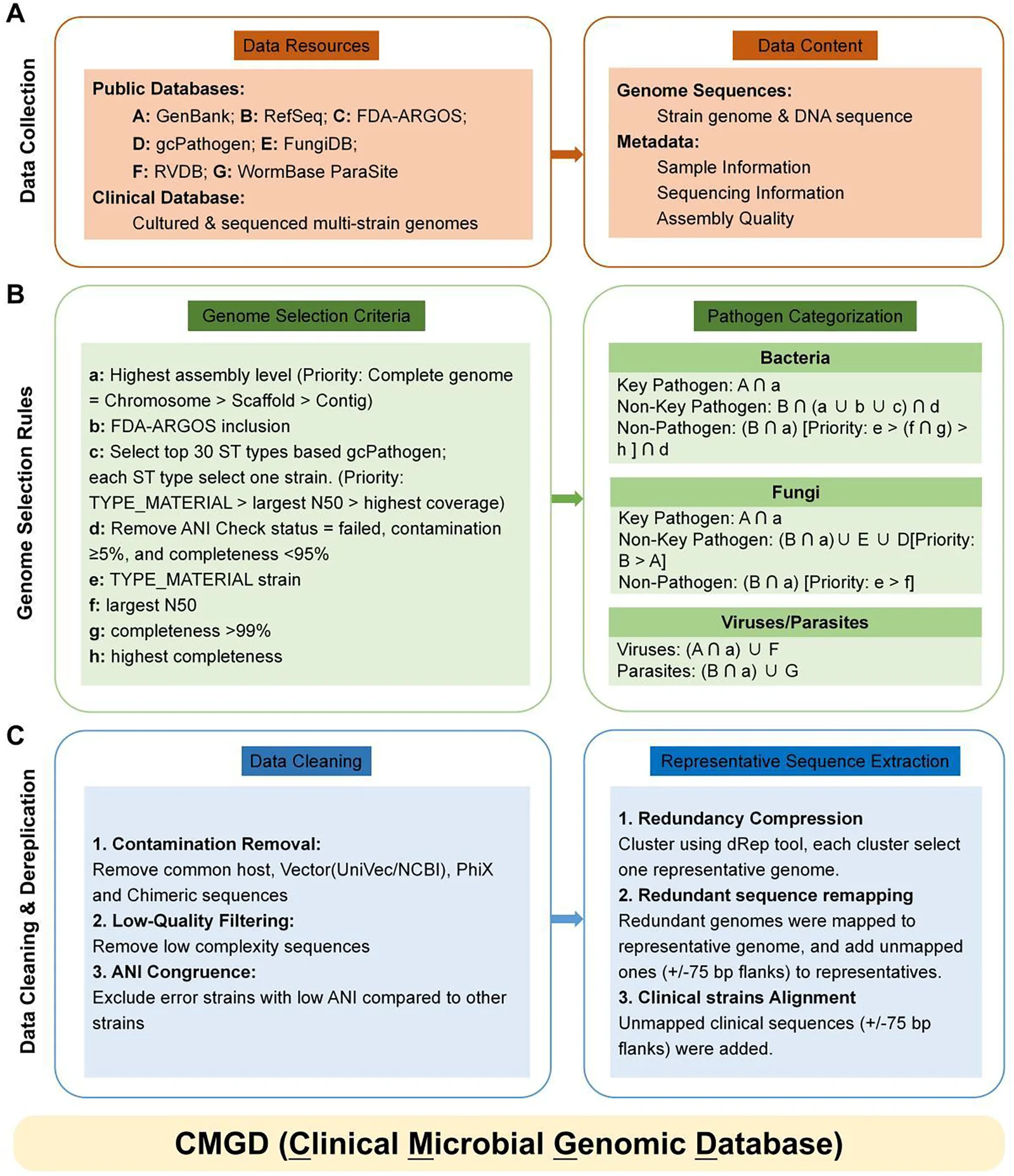
Schematic overview of CMGD construction. **(A)** Sources of microbial genome strains and sequences. **(B)** Sequence inclusion criteria for pathogens stratified by pathogen type and clinical priority level. **(C)** Workflow for microbial sequence curation and redundancy reduction.

Bacteria and fungi were stratified into three tiers based on clinical importance and their representation in recognized pathogen databases (FDA-ARGOS, gcPathogen, and FungiDB). Key pathogens represented clinically high-priority species. When classifications differed across sources, the higher clinical-priority category took precedence (key pathogen > non-key pathogen > non-pathogen). Species not meeting the key-pathogen criteria but catalogued in at least one recognized pathogen database were classified as non-key pathogens, whereas species meeting neither criterion were classified as non-pathogens. Emerging or reclassified taxa are reassessed during scheduled CMGD updates according to updated database, literature, and epidemiological evidence, with tier assignments revised when appropriate. Specific inclusion rules were applied to each group (**Figure 1B**). For key bacterial and fungal pathogens, strains with the highest assembly level (Complete = Chromosome> Scaffold > Contig) were prioritized from GenBank. For non-key bacterial pathogens, strains at the highest assembly level, FDA-ARGOS entries, and the top-30 sequence-type (ST) strains from gcPathogen sourced from RefSeq were included. For non-key fungal pathogens, RefSeq strains with the highest assembly level together with strains from FungiDB and gcPathogen were included. For non-pathogenic bacteria and fungi, only a single representative RefSeq strain at the highest assembly level was retained. For viruses, GenBank entries with the highest assembly level and the entirety of the RVDB sequences were incorporated. For parasites, RefSeq strains at the highest assembly level together with all sequences in WormBase ParaSite were included.

Included genomic sequences were processed using BLAST (v2.11.0+) (McGinnis and Madden, 2004) to remove common host sequences, vector sequences, PhiX sequences and chimeric sequences, and low-complexity regions were masked using dustmasker (v1.0.0) (Morgulis et al., 2006; Chrisman et al., 2022; Chorlton, 2024) (**Figure 1C**). Genomic similarity between strains was assessed using dRep (Olm et al., 2017), and outlier strains defined by low average nucleotide identity (ANI) to all others were excluded. dRep was further utilized for clustering (--ignoreGenomeQuality -pa 0.99 --cov_thresh 0.1 --SkipSecondary), with one representative strain selected from each cluster to form the core microbial database.

To ensure comprehensive coverage, the remaining (non-representative) strain sequences within each dRep cluster were cross-aligned against the representative genomes. Regions flanking unaligned segments (± 75 bp) were extracted to capture accessory genomic elements unique to non-representative strains and appended to the representative genome sequences. Clinical isolate sequences and RVDB-derived DNA sequences were then aligned to the database. Non-redundant sequences (>95% nucleotide identity over >90% sequence length) were integrated into the final collection. This operational dereplication criterion is consistent with thresholds used in published microbial gene catalogues and was applied to reduce highly similar sequence fragments (Karlsson et al., 2014; Mende et al., 2020; Commichaux et al., 2021). Finally, database was indexed using “makeblastdb” and “BWA index” for subsequent BLAST and BWA alignments, respectively (McGinnis and Madden, 2004; Li, 2013). The resulting database used in this study was designated CMGD v1.0 and comprised 28,164 representative strains and 433,737 DNA sequences. CMGD v1.0 has also been implemented in an online workflow for automated analysis of single-end short-read mNGS FASTQ data.

### Sequencing data quality control and host depletion

Raw sequencing reads were processed using Trimmomatic (v0.40) to remove adapter sequences and low-quality bases with parameters of “LEADING:3 TRAILING:3 SLIDINGWINDOW:4:15 MINLEN:36” (Bolger et al., 2014). To minimize interference from human DNA, Bowtie2 (v2.4.2) was used to align reads against host reference genomes (Langmead and Salzberg, 2012). Only unaligned reads were retained for subsequent microbial analysis. Three reference configurations were employed to evaluate the host depletion efficiency: GRCh38, T2T - CHM13 (Nurk et al., 2022), and a combined GRCh38-T2T reference.

### Data alignment and microbial taxonomic identification

Microbial taxonomic classification was performed using Kraken2, BLAST, and BWA algorithms. BWA (v0.7.15-r1140) mem algorithm was used to align reads to CMGD with parameters (-m 100 -T 30) (Li, 2013). Alignments were filtered based on the following criteria: (i) alignment score exceeding the minimum of 80% read length or 60, or (ii) the longest match segment (M) in the CIGAR string exceeding 90% score criteria. BLASTn (v2.11.0+) with the megaBLAST option (evalue = 1×10^-10^, word_size = 28, max_target_seqs = 10, max_hsps = 1) was used to align reads to CMGD (BLAST-CMGD) and the NCBI NT database (2023-12, BLAST-NT) which contained the reference microbial sequences from archaea, bacteria, viruses and fungi (McGinnis and Madden, 2004). Kraken2 (v2.1.3) was used to classify reads against the Kraken2 PlusPF prebuilt database (k2_pluspf_20240112) (Wood et al., 2019). The number of total aligned reads and uniquely aligned reads were calculated independently for each identification algorithm. Total aligned reads were categorized as unique alignments if they mapped exclusively to a single species, whereas those aligning to multiple taxa simultaneously were defined as multi-mapped reads. For BWA- and BLAST-based analyses, alignment rate refers to the proportion of reads aligned to the target species, unique alignment rate to the proportion aligned exclusively to a single species, and erroneous alignment rate to the proportion assigned to non-target species. For Kraken2, the corresponding outputs are described as species-level, genus-level, and erroneous classification rates.

### In silico data simulation

To benchmark host subtraction efficiency and microbial detection sensitivity across database configurations, both host and microbial reads were simulated. Human (host) reads were simulated from NT-derived *Homo sapiens* sequences (taxid: 9606) at five sequencing depths (1, 5, 10, 20, and 30 million reads), with 20 replicates per depth. For microbial performance evaluation, 65 representative species (27 bacteria, 12 fungi, 16 viruses, 8 parasites, and 2 archaea) were each simulated with varying read counts (15, 50, 100, 500, 1,000, 5,000, 10,000, 50,000, and 100,000 reads), with 20 replicates per condition. For each of the 65 representative species, 10 strains were randomly sampled for simulation, with preference for higher assembly completeness (Complete/Chromosome > Scaffold > Contig). Across all 65 simulated species, 245 of 2,672 unique genome accessions (9.17%) exactly matched representative genomes retained in CMGD, whereas 90.83% were not exact representative-genome matches (**Supplementary Table S1**).

To evaluate species-level discrimination, three pairs of genetically similar and clinically confounding microorganisms (*E. coli*/*S. flexneri*, *S. aureus*/*S. epidermidis*, and *C. neoformans*/*C. gattii*) were simulated at ratios of 1:0, 0:1, 1:1, 1:3, 3:1, 1:10, and 10:1, with 10 replicates per ratio. All reads were simulated using Wgsim (v0.3.2, https://github.com/lh3/wgsim) with an error rate of 0.001 and a read length of 75 bp (Li et al., 2009).

### Clinical specimen collection and mock sample preparation

All clinical specimens enrolled in this study were collected from Jinan Central Hospital, with Ethics Committee of Jinan Central Hospital approval number of 20260623003. Confirmed negative samples by both CMT and mNGS were used as the matrix. Positive mock samples were prepared by spiking pathogen-free CSF specimens with genomic DNA from clinically isolated *S. aureus* and *S. epidermidis* strains at predefined ratios (1:0, 0:1, 1:3, 3:1, and 1:1). Each condition was set up in three replicates, yielding a total of 15 positive mock samples. Clinical specimens including blood, bronchoalveolar lavage fluid, intervertebral disc tissue, and other types, that yield positive results confirmed by both CMT and mNGS were enrolled from June 2023 to January 2026.

### mNGS library preparation and sequencing

The nucleic acids were extracted using the Nucleic Acid Extraction Reagent Kit (YGZZ014-48, HugoBiotech, Beijing, China), and sequencing libraries were constructed using the Universal Sequencing Reaction Kit (YGZZ005, HugoBiotech, Beijing, China). The library concentration and integrity of the libraries were measured using Qubit 4.0 (Thermo Fisher Scientific, USA) and Agilent A2100 Bioanalyzer (Agilent Technologies, USA). Libraries passing quality control were sequenced on the Illumina 550DX platform (Illumina, Inc., USA) with sequencing model of single-end 75 bp.

### Computational resource for CMGD running

In this study, all computational analyses were carried out on a Linux server with 64 CPU cores and 256 GB RAM. The minimum hardware requirements for running the database and workflow are a server with at least 16 threads and 48 GB RAM. For microbial species-level analysis under the minimum computational resource, annotation took approximately 5 minutes for samples containing 10 million 75-bp reads and 20 minutes for samples with 50 million 150-bp reads. BLASTn was run with 16 threads for runtime benchmarking, and CPU time was captured using the Linux time utility. Database construction and indexing durations were not included in the reported CPU-time values.

### Statistical analysis

All statistical analyses were conducted using R (v4.1.0). The Wilcoxon rank-sum test was used to compare detection metrics between different analytical methods. For comparisons among the three host-reference strategies, the Kruskal-Wallis test was used to assess overall differences, followed by pairwise Wilcoxon rank-sum tests. *P* values for pairwise comparisons were adjusted using the Holm method. A linear mixed-effects model was fitted to evaluate species read counts between BLAST-CMGD and BLAST-NT approaches in clinical specimens (Bates et al., 2015), with detection method as the fixed effect and sample identity together with the sample-by-species interaction as random effects, to account for baseline differences across samples and heterogeneity in species composition. Agreement between BLAST-CMGD and BLAST-NT was further evaluated using Bland-Altman analysis of log10-transformed paired read counts and Lin’s concordance correlation coefficient (CCC). Statistical significance was defined as *P* < 0.05, with values below 0.001 reported as *P* < 0.001.

## Results

### CMGD database composition and characteristics

In this study, the standard analysis framework for clinical mNGS data integrates host-component depletion with pathogen detection based on CMGD, constructed using a tiered stratification strategy (**Figure 1**). Initially, a comprehensive catalog of clinically relevant microorganisms was curated from multiple pathogen databases (see Materials and Methods), encompassing 14,327 bacteria, 486 archaea, 1,031 fungi, 338 parasites, and 2,159 viruses (**Figure 2A**). **Figure 2B** summarizes the strain and sequence counts of collected microorganisms from RefSeq, GenBank, FungiDB, gcPathogen, FDA-ARGOS, WormBase ParaSite, and RVDB. Microorganisms were subsequently categorized into three tiers, key pathogens, non-key pathogens, and non-pathogens based on their taxonomic classification, clinical significance, and database availability. We implemented differentiated collection criteria for each tier, “exhaustive inclusion” for key pathogens to ensure maximum sensitivity, “prioritized inclusion” for non-key pathogens derived from RefSeq, FungiDB, gcPathogen, and FDA-ARGOS, and “representative inclusion” for non-pathogenic species (see Materials and Methods; **Figure 1**). This systematic approach yielded an initial dataset of 84,251 strains and 884,347 DNA sequences (primarily from RVDB). To optimize computational efficiency for clinical diagnostics, we performed redundancy reduction, ultimately retaining a refined collection of 28,164 representative strains and 433,737 high-quality DNA sequences (**Figure 2D**).

**Figure 2:**
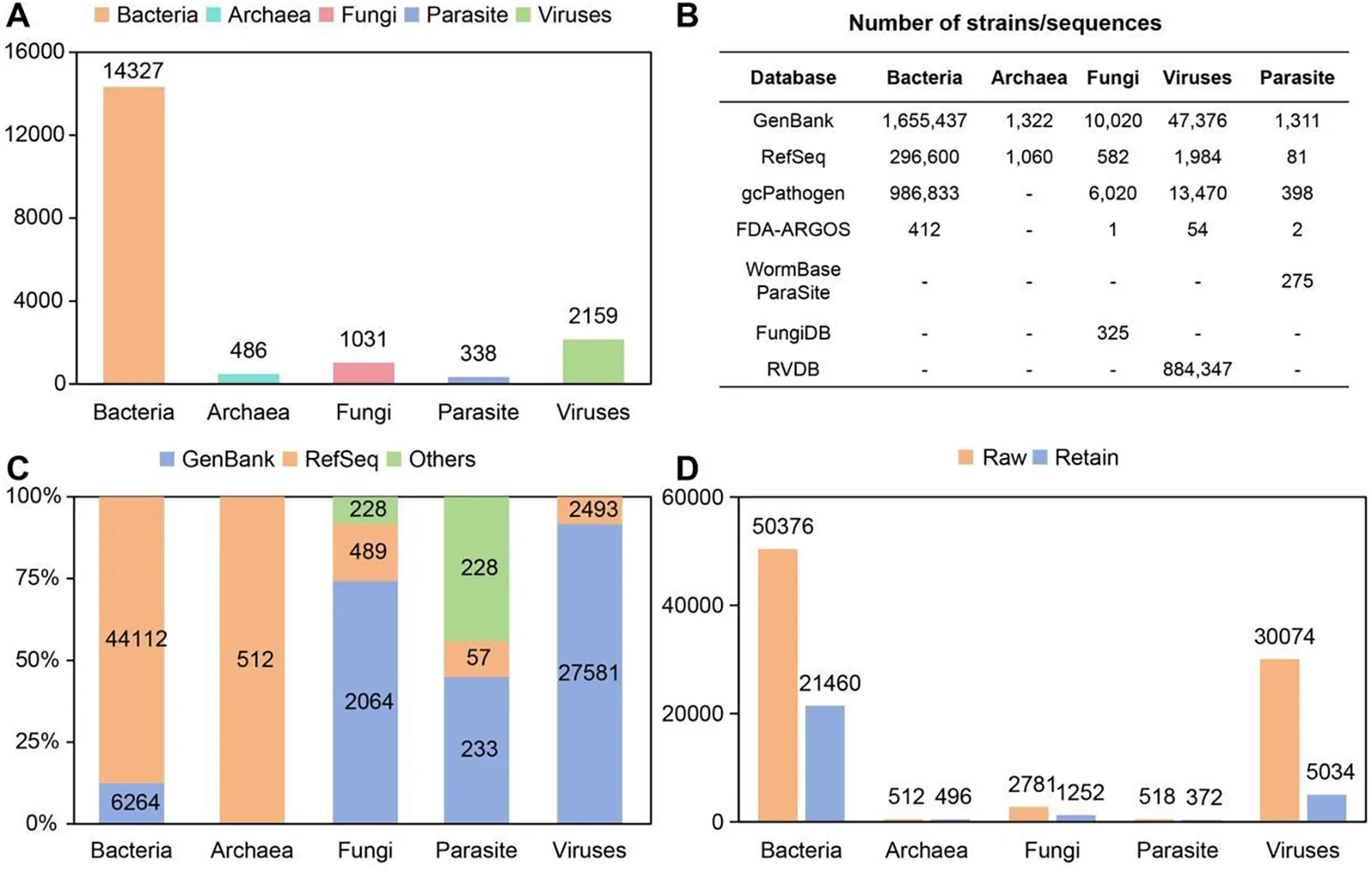
Composition and source distribution of CMGD. **(A)** Numbers of bacterial, fungal, archaeal, parasitic, and viral species included in the database. **(B)** Numbers of strains and DNA sequences derived from each source database. **(C)** Strain counts stratified by inclusion rule and pathogen type after tiered filtering. **(D)** Representative strain counts after curation and de-duplication.

### Host depletion performance on simulated datasets

Because host depletion is a prerequisite for accurate pathogen detection, we first evaluated the performance of different human reference genomes on simulated datasets. A single human reference genome may leave host-derived sequences insufficiently removed, whereas combining multiple human references may increase the risk of losing microbial reads. To assess this balance, we compared three host depletion strategies, namely GRCh38 alone, T2T alone, and a combined GRCh38-T2T reference using simulated host and microbial datasets. Host sequences were simulated at depths of 1M, 5M, 10M, 20M, and 30M reads, while microbial sequences were simulated for 65 representative species at concentrations ranging from 15 to 100K reads. For simulated human sequences, the combined GRCh38-T2T strategy achieved a significantly higher depletion rate (99.775%) compared with T2T alone (99.731%) and GRCh38 alone (99.593%) (pairwise Wilcoxon rank-sum tests, all Holm-adjusted *P* < 0.001; **Figure 3A**). For simulated microbial reads, the proportion of reads erroneously removed as host reads was highest in GRCh38-T2T (0.591%), followed by T2T (0.571%) and GRCh38 (0.565%), although these differences were not statistically significant (Kruskal-Wallis test, *P* = 0.322; all pairwise Wilcoxon rank-sum tests, P > 0.05; **Figure 3B**). Consequently, the GRCh38-T2T strategy was adopted to maximize host removal while preserving microbial signals for subsequent analyses.

**Figure 3:**
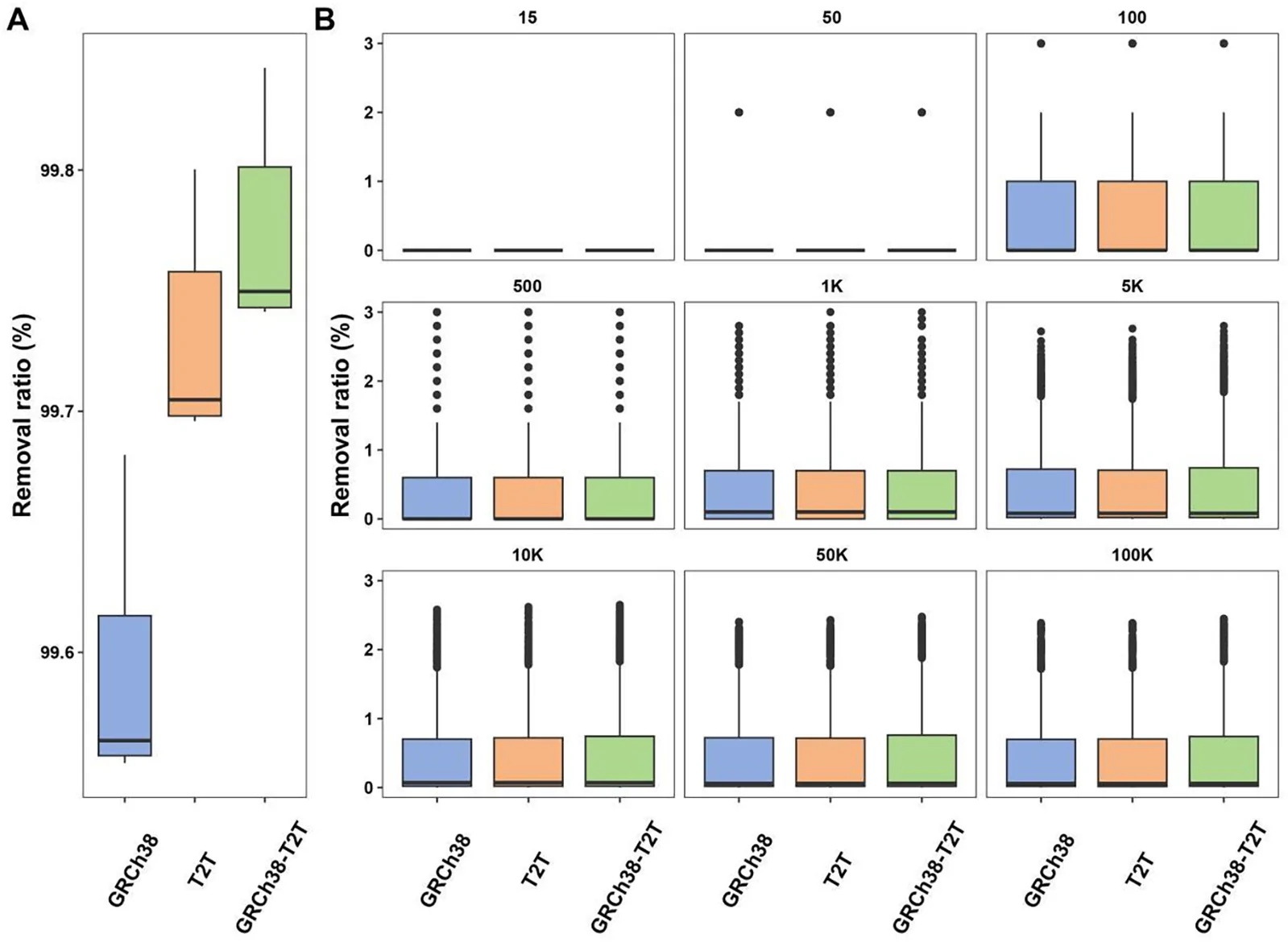
Host read removal performance in simulated datasets. **(A)** Host read removal efficiency for simulated human reads using GRCh38, T2T, and GRCh38-T2T reference genomes. **(B)** Proportions of simulated microbial reads erroneously removed as host-derived reads at varying sequencing depths using the three reference configurations.

### Taxonomic identification performance on simulated data

With the host depletion strategy established, we next evaluated CMGD’s taxonomic identification performance against the broadly utilized standard Kraken2 database, implementing detection via both BLAST-CMGD (BLAST) and BWA-CMGD (BWA) alignment pipelines to assess aligner-dependent effects.

For the host removal simulated host datasets, the proportion of reads unexpectedly and uniquely aligned to microbes followed the order BWA-CMGD > BLAST-CMGD > Kraken2 (**Supplementary Figure S1A**). The majority of these sequences were erroneously assigned to Human betaherpesvirus 6 (HHV-6) and the proportion misaligned to other species remained extremely low, with BWA-CMGD yielding the lowest rate (**Supplementary Figures S1B, C**). Tracing the source of these residual human sequences revealed that most originated from records annotated as “Homo sapiens isolate … endogenous virus endogenous *human herpesvirus 6* sequence” carrying taxid 9606, likely a consequence of systematic mis-annotation in the NT database. Both BWA-CMGD and BLAST-CMGD methods correctly identified over 95% of these endogenous sequences, whereas Kraken2 identified below 90% (**Supplementary Figure S1D**).

We next evaluated the identification performance of each method on simulated microbial datasets. For the 65 representative simulated species, the target-species alignment rate with BWA-CMGD ranged from 93.9% to 100%, with unique alignment rates ranging from 0.017% to 99.61% and erroneous alignment rates ranging from 0 to 0.58% (**Figure 4**). In contrast, BLAST-CMGD showed greater variability in target-species alignment rate (46.3–99.98%) and erroneous alignment rate (0–13.4%). Notably, 21.6% (n=14) of the 65 simulated species were absent from the Kraken2 database, including *Brucella neotomae*, Human alphaherpesvirus *2* (HSV-2), and several fungi and parasites. Among the 51 species present in Kraken2, genus-level classification rates of simulated species ranged from 14.45% to 99.94%, species-level rates from 0.69% to 99.91%, and erroneous rates from 0% to 1.18%. For the 14 absent species, erroneous classification to other taxa ranged from 0.028% to 52.08% (**Figure 4C**).

**Figure 4:**
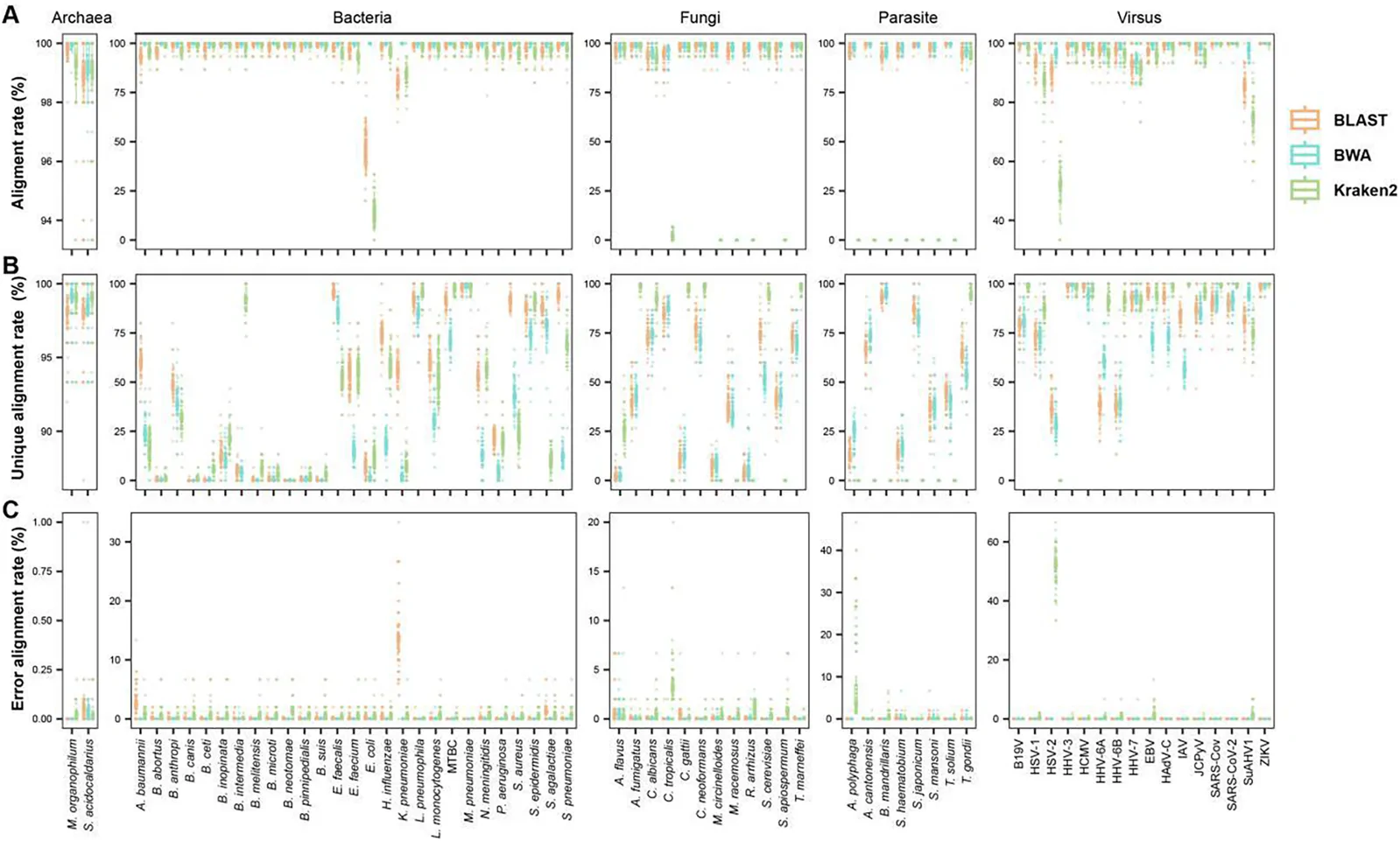
Taxonomic assignment metrics for simulated microbial reads. **(A)** Target-species alignment/classification rate, **(B)** unique species-level assignment rate, and **(C)** erroneous assignment rate for each simulated species under the BLAST-CMGD, BWA-CMGD, and Kraken2 workflows. For BWA and BLAST, these metrics are based on sequence alignment; for Kraken2, they represent k-mer-based taxonomic classification.

Database coverage differed substantially between CMGD and the standard Kraken2 database: 14 of the 65 simulated species (21.6%) were not represented in the Kraken2 database, whereas all evaluated species were represented in CMGD. For these 14 absent species, Kraken2 erroneous classification to other taxa ranged from 0.028% to 52.08%, indicating the effect of reference-database coverage on classification performance. Among the 51 species represented in both databases, the two workflows showed different performance profiles. Kraken2 yielded higher species-level unique assignment rates for 42 species, whereas BWA-CMGD yielded higher unique assignment rates for the remaining nine species. In contrast, BWA-CMGD showed lower erroneous assignment rates. These findings indicate that database composition and classification strategy contributed separately to the observed performance differences. For *Escherichia coli* and *Klebsiella pneumoniae*, which showed atypical results in BLAST-CMGD analyses (**Figure 4**), increasing the “-max_target_seqs” parameter significantly improved performance (**Supplementary Figure S2**). However, the analysis time and computational resource consumption also increased substantially (**Supplementary Figure S3**).

### Species-level discrimination of closely related pathogens

Accurate species-level identification of closely related pathogens is critical in clinical mNGS, as members of the same genus frequently encompass both commensal colonizers and true pathogens, and misidentification may precipitate inappropriate antimicrobial therapy with adverse clinical consequences. To evaluate the discriminatory power of CMGD for closely related species, the BWA-CMGD approach was applied to simulated reads from three confounding species pairs. For *Cryptococcus neoformans* and *Cryptococcus gattii* pair, *C. neoformans* achieved a high target-species alignment rate of 99.5% with a unique alignment rate of 70.5%, while 5.5% of its reads cross-aligned to *C. gattii.* Conversely, *C. gattii* showed a target-species alignment rate of 99.98% and a unique alignment rate of 13.0%, with 11.4% of its reads co-aligning to *C. neoformans* (**Figure 5A**). For *Staphylococcus aureus* and *Staphylococcus epidermidis* pair, *S. aureus* reached a target-species alignment rate of 99.94% with a 42.2% unique alignment rate, whereas *S. epidermidis* exhibited a higher unique alignment rate of 74.4%. Cross-alignment accounted for 2.3% of *S. aureus* reads mapping to *S. epidermidis* and 7.8% of *S. epidermidis* reads mapping to *S. aureus* (**Figure 5B**). The most pronounced sequence homology was observed between *E. coli* and *Shigella flexneri*. Despite a target-species alignment rate of 99.98%, *E. coli* reads yielded an extremely low unique alignment rate of only 0.14%, with 94.1% of reads aligning to *S. flexneri*. Similarly, 99.19% of *S. flexneri* reads co-aligned to *E. coli*, resulting in a unique alignment rate of merely 0.029% (**Figure 5C**). The extremely low unique alignment rates reflect near-complete genomic identity at the nucleotide level rather than any analytical deficiency of CMGD. Accordingly, these read-assignment metrics should be interpreted as analytical evidence rather than fixed clinical reporting thresholds, and species-level assignment is not considered reliable for such highly homologous taxa when discriminatory sequence evidence is insufficient.

**Figure 5:**
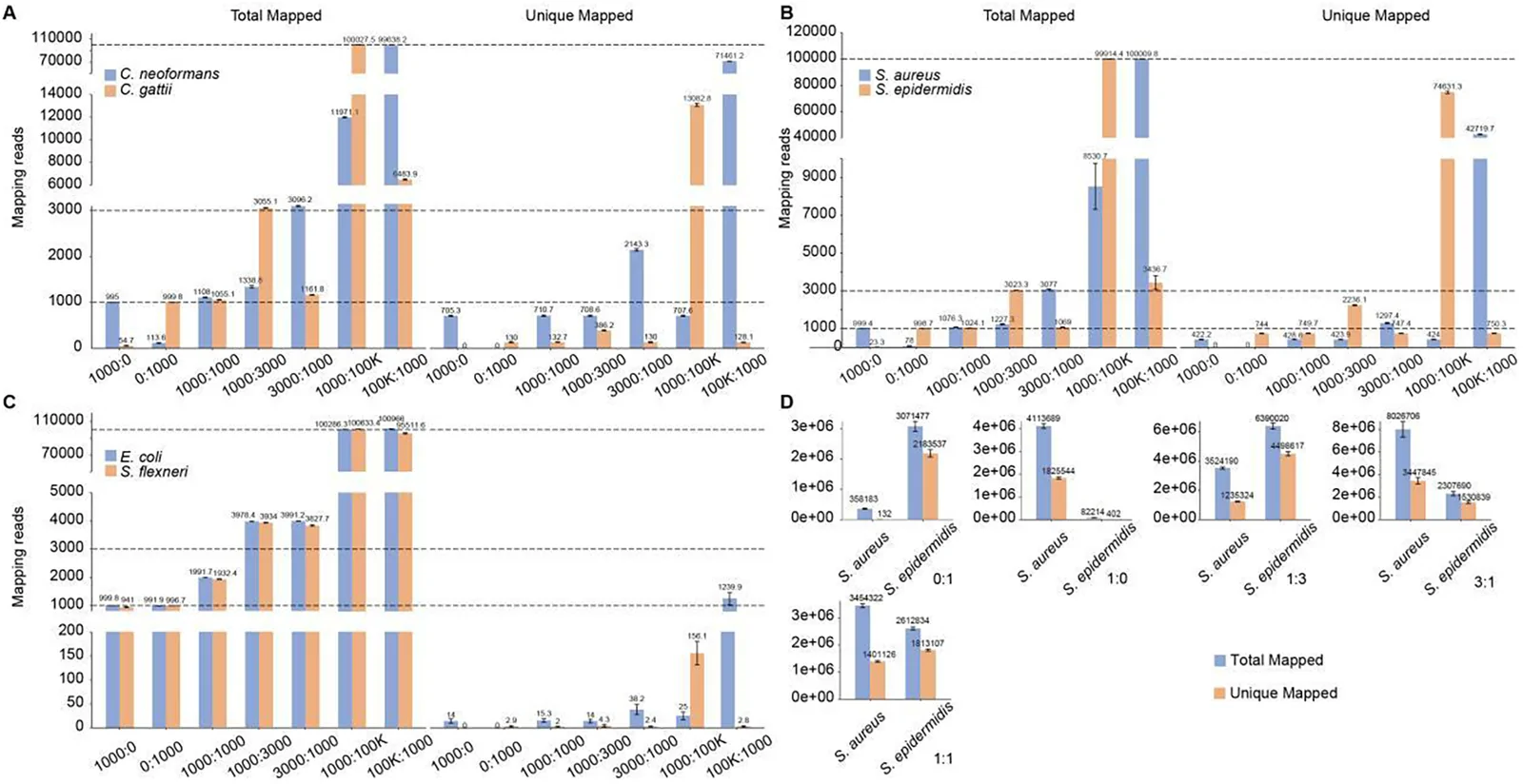
Target-species alignment in closely related species pairs. Detection outcomes at various mixing ratios for *C. neoformans* and *C. gattii* **(A)**, *S. aureus* and *S. epidermidis* **(B)**, and *E. coli* and *S. flexneri* **(C)**. Colors represent distinct species, while the left and right panels display total and unique alignment read counts, respectively. **(D)** Mock sample validation results for *S. aureus* and *S. epidermidis* at mixing ratios 0:1, 1:0, 1:3, 3:1, and 1:1. Total mapped reads and uniquely mapped reads are shown in blue and yellow, respectively.

To further validate the quantitative performance of CMGD in complex mixtures, a spike-in experiment was conducted using *S. aureus* and *S. epidermidis* DNA mixed at five defined ratios (0:1, 1:0, 1:3, 3:1, and 1:1) in pathogen-negative cerebrospinal fluid (CSF). The observed ratios of species-specific unique reads (6.05×10^-5^:1, 1:0.00022, 2.25:1, 1:3.64, and 1:1.29) were broadly consistent with the expected input proportions (**Figure 5D**), demonstrating that CMGD can accurately reflect relative pathogen abundance even in compositionally complex clinical matrices. The residual non-zero signal detected under single-species conditions is attributable to background cross-alignment or inherent sequencing noise, rather than true existence.

Collectively, these findings demonstrate that while closely related species can achieve high alignment accuracy to their respective reference genomes, the pervasive cross-alignment, particularly between *E. coli* and *Shigella*, renders unique alignment rate alone insufficient for definitive species-level identification in clinical metagenomic diagnostics. The spike-in validation further confirms that CMGD provides reliable quantitative discrimination between co-occurring species, supporting its utility in complex polymicrobial clinical scenarios.

### Performance evaluation on public clinical datasets

Having established the analytical performance of CMGD on simulated datasets, we next evaluated its performance using six previously published clinical cohorts comprising 398 samples (Wilson et al., 2018; Saha et al., 2019; Gu et al., 2021; Guo et al., 2021; Hong et al., 2022; Tang et al., 2022). Positive samples were defined as those with at least one pathogen reported by CMT, mNGS, or other microbiological or clinical diagnostic assessments in the original studies. This cohort included 296 positive samples predominantly derived from lung tissue (35.47%) and CSF (26.01%) (**Supplementary Figure S4**). A total of 154 species were reported, including 117 bacteria, 21 fungi, 15 viruses, and 1 parasite. The most frequently reported pathogens were *Aspergillus fumigatus* (n=39), *Streptococcus pneumoniae* (n = 31), *S. aureus* (n = 30), *E. coli* (n = 29) and Human Herpesvirus 4 (HHV-4 or EBV, n=21) (**Supplementary Figure S4C**). Detailed information on the six public clinical cohorts is provided in **Supplementary Table S2**. Among the 512 pathogen detections reported in the original studies, 16.8% (n = 86) were concordantly identified by CMT, mNGS, and the source-study reference/diagnostic assessment. Notably, 52.3% were reported exclusively by mNGS, indicating a higher detection yield than CMT and the source-study reference/diagnostic assessment (**Figure 6A**).

**Figure 6:**
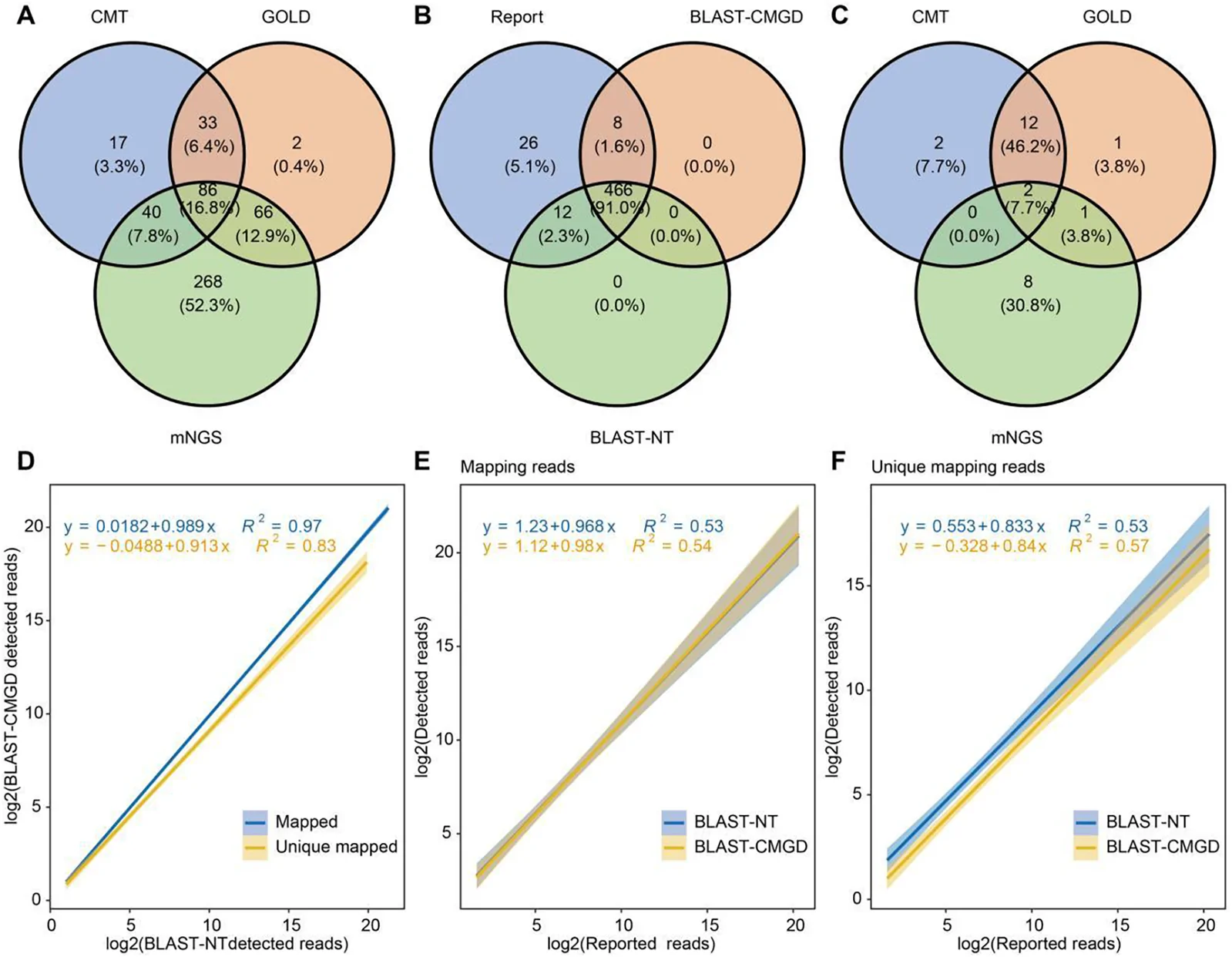
CMGD performance on public clinical datasets. **(A)** Venn diagram illustrating overlap among pathogen detections reported by CMT, mNGS, and source-study reference/diagnostic assessments across the 296 positive samples. **(B)** Concordance among literature-reported positive organisms, BLAST-CMGD results, and BLAST-NT results. **(C)** Literature-based identification status of organisms undetected by both BLAST-CMGD and BLAST-NT. **(D)** Correlation between BLAST-NT and BLAST-CMGD sequence counts for co-detected species. Blue represents total mapped reads, while yellow denotes unique mapped reads. **(E)** Correlation between literature-reported read counts and total mapped read counts detected by BLAST-NT and BLAST-CMGD. **(F)** Correlation between literature-reported read counts and uniquely mapped read counts detected by BLAST-NT and BLAST-CMGD.

To comparatively evaluate CMGD performance, BLAST-NT results against the NCBI NT database were used as a computational comparator. Detection concordance between the BLAST-NT and BLAST-CMGD pipelines was observed for 91.0% of the evaluated pathogen detections (n = 466). Only a small fraction of species (3.9%) were exclusively detected by either BLAST-CMGD (1.6%) or BLAST-NT (2.3%) methods while 5.1% (n=26) remained undetected by both approaches (**Figure 6B**). For pathogens identified by both databases the correlation of total detected reads was extremely high (R^2^ = 0.97), and the uniquely aligned reads demonstrated a strong correlation as well (R^2^ = 0.83) (**Figure 6D**). Both BLAST-NT and BLAST-CMGD results showed comparable correlations with the original literature reports in terms of total aligned reads and uniquely aligned reads (**Figures 6E, F**).

Among the 12 detections (2.3%) exclusive to BLAST-NT, 9 involved unclassified species designated as “.sp” that were absent from the CMGD database. The remaining 3 cases yielded only 1, 1, and 59 multi-mapping reads by BLAST-NT. Importantly, these specific pathogens were originally reported based on CMT or other source-study diagnostic evidence, rather than by mNGS. Among the 26 detections undetected by both databases, 15 were not reported by mNGS in the original literature (**Figure 6C**). Of the 11 pathogens reported by mNGS, two were also supported by both CMT and the source-study reference/diagnostic assessment, one was additionally supported by the source-study reference/diagnostic assessment, and eight were reported exclusively by mNGS (**Figure 6C**). To investigate the clinical significance of the 11 undetected pathogens, we performed a re-analysis using the online-NCBI-BLAST-tool with default parameters (max_target_seqs = 100, word_size = 28). Only four hits with extremely low read counts were retrieved, with read counts of 1, 2, 8, and 16 reads, respectively. All of these hits represented non-specific sequence matches (**Supplementary Figure S5**).

### Evaluation on internal clinical samples

Finally, we evaluated CMGD using an additional 30 retrospectively selected internal clinical mNGS-positive samples. BLAST-CMGD identification yielded a significantly higher number of detected species than BLAST-NT (*P* < 0.001; **Figure 7A**). Among mNGS-reported positive species, read counts obtained using BLAST-CMGD and BLAST-NT were strongly correlated for both total mapped reads and uniquely mapped reads, with R² values of 0.99 and 0.89, respectively (**Figures 7B, C**). Additional agreement analysis showed a Bland-Altman bias of 0.07 log10 for total mapped reads (95% CI, 0.03–0.11; 95% LoA, −0.17 to 0.31), corresponding to an NT/CMGD ratio of 1.18 (95% CI, 1.08–1.29), with a CCC of 0.99. For uniquely mapped reads, the bias was 0.24 log10 (95% CI, 0.11–0.36; 95% LoA, −0.51 to 0.98), corresponding to an NT/CMGD ratio of 1.73 (95% CI, 1.30–2.29), with a CCC of 0.92. For all species co-detected by CMGD and NT, including laboratory background contaminants, components of human microbial flora, and true pathogens, significant positive correlations were also observed for total mapped reads (R² = 0.89) and uniquely mapped reads (R² = 0.80). Bland-Altman analysis yielded biases of −0.07 log10 for total mapped reads (95% LoA, −1.01 to 0.86; CCC = 0.82) and 0.02 log10 for uniquely mapped reads (95% LoA, −0.80 to 0.85; CCC = 0.89), indicating greater species-level variability (**Supplementary Figures S6A, C**). Given the strong sample- and species-specific effects on microbial read counts, a mixed-effects model was further employed to assess overall differences between the two databases. This analysis revealed no statistically significant difference in total mapped read counts between CMGD and NT (*P* = 0.1987; **Supplementary Figure S6B**), whereas uniquely mapped read counts differed significantly between the two approaches (*P* = 0.0458; **Supplementary Figure S6D**).

**Figure 7:**
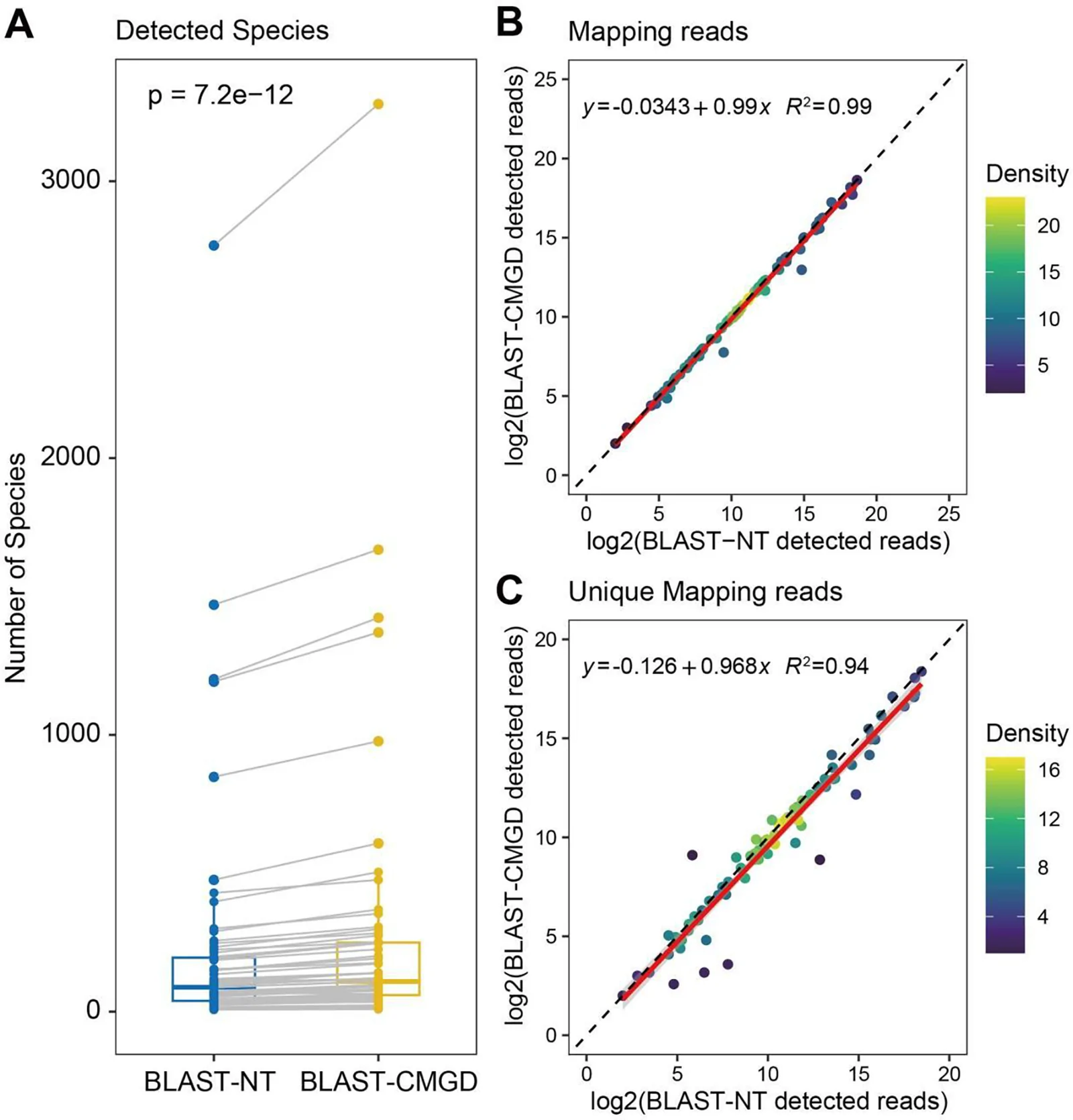
CMGD performance on retrospective clinical samples. **(A)** Number of species identified by BLAST-CMGD and BLAST-NT. **(B, C)** Correlation between CMGD and NT for mNGS-reported positive species, based on **(B)** total mapped reads and **(C)** uniquely mapped reads.

## Discussion

In this study, we developed and systematically evaluated a clinically oriented mNGS framework based on CMGD, a curated repository encompassing over 18,000 microbial species. Unlike conventional reference databases that primarily aim to maximize taxonomic breadth, CMGD was designed using a tiered clinical stratification strategy, in which clinically important pathogens were exhaustively included, non-key pathogens were selectively prioritized, and non-pathogenic organisms were represented in a reduced manner, thereby balancing diagnostic sensitivity, taxonomic specificity, and computational feasibility. By jointly optimizing host depletion, database curation, and alignment strategy, this study provides a practical framework for improving the accuracy and interpretability of clinical mNGS workflows.

Host depletion represents a critical upstream determinant of mNGS diagnostic performance, as clinical specimens are usually dominated by human-derived reads. Inadequate host removal reduces effective microbial sequencing depth, whereas overly aggressive host depletion may inadvertently remove microbial reads with human-like sequences. Although Bowtie2 is widely used in clinical mNGS workflows (Diao et al., 2025), host depletion performance is also strongly dependent on the completeness of the human reference genome. In this study, a comparison of different host reference strategies showed that the combined GRCh38 and T2T reference achieved the highest host depletion rate compared with either GRCh38 or T2T alone, reaching 99.775%, while the proportion of microbial reads erroneously removed as host reads was not significantly increased. The advantage of a multi-genome host depletion strategy is likely attributable to the complementary coverage of different human genome assemblies, which reduces the blind spots inherent to any single reference genome. A similar principle also applies to microbial reference database construction. Microbial species are not genetically uniform entities, but usually consist of a conserved core genome and a variable accessory genome, collectively referred to as the pan-genome (Van Rossum et al., 2020). Therefore, a single representative genome is unlikely to capture the full genomic diversity of a species, particularly for bacteria with extensive strain-level variation, horizontal gene transfer, plasmids, virulence islands, or antimicrobial resistance elements, a consideration that directly informed the tiered construction strategy of CMGD.

An important observation in this study was that residual human reads retained after host depletion could still be aligned to microbial references, particularly HHV-6-related sequences (**Supplementary Figure S1**). The tracing analysis showed that many of these reads originated from sequences annotated as “Homo sapiens isolate … endogenous virus endogenous human herpesvirus 6 sequence” with taxid 9606, suggesting that some apparent microbial hits are in fact human-associated endogenous viral elements or misannotated entries. This finding indicates that false microbial signals in mNGS may arise not only from insufficient host depletion or laboratory contamination, but also from misannotated, contaminated, or ambiguously classified sequences in public nucleotide repositories. Such taxonomic mis-annotations are widespread in NCBI repositories, affecting approximately 3.6% of prokaryotic genomes in GenBank and 1% of those in RefSeq (Ciufo et al., 2018; Lupo et al., 2021; Chorlton, 2024). These errors may lead to false-positive detections and distorted abundance estimates when unfiltered public resources are used directly without quality control. Thus, the residual host-read analysis supports the need for curated, clinically focused, and quality-controlled reference databases such as CMGD. As part of future database update strategy, systematically collecting, curating, and excluding mis-annotated sequences during database construction would represent a practical approach. This strategy would mitigate the impacts of annotation errors in subsequent CMGD releases.

The comparison between CMGD-based methods and Kraken2 highlights the complementary effects of database coverage and classification strategy on taxonomic performance. Fourteen of the 65 simulated species (21.6%) were absent from the standard Kraken2 database used in this study, indicating that reference coverage directly determines the range of detectable taxa and contributes to workflow-level differences. Among the 51 species represented in both databases, Kraken2 achieved higher species-level unique assignment rates for 42 taxa, whereas BWA-CMGD showed lower erroneous assignment rates overall. This contrast reflects their different analytical designs: Kraken2 enables rapid hierarchical classification through k-mer matching, while CMGD supports alignment-based analysis with BWA and BLAST. Thus, the two workflows offer complementary strengths: Kraken2 provides efficient species-level classification for well-represented taxa, whereas BWA-CMGD combines broader reference coverage with more conservative taxonomic assignment. These findings underscore the importance of jointly considering database completeness, assignment accuracy, and computational efficiency when selecting an clinical mNGS analysis workflow.

Although BLAST is a widely used high-sensitivity sequence-alignment comparator, it is computationally expensive and slow for routine clinical throughput, especially when querying large databases such as NT. In addition, BLAST performance is also sensitive to parameter settings. Notably, the “max_target_seqs” parameter substantially influences both alignment quality and speed, especially for closely related species, because the algorithm preferentially returns the top indexed hits rather than the most biologically relevant matches. In this study, the “max_target_seqs” parameter substantially influenced the alignment accuracy of *E. coli* and *K. pneumoniae*. At a “max_target_seqs” value of 10, only 50%-80% of simulated *E. coli* and *K. pneumoniae* reads correctly aligned to the target species (**Supplementary Figure S2**), whereas increasing this parameter to 100 and 500 improved accuracies, albeit at the cost of significantly longer runtime. Therefore, although BLAST is valuable for resolving ambiguous cases, its computational demands and parameter sensitivity limit its use as the primary high-throughput alignment method in this workflow. In contrast, BWA has been widely adopted for pathogen identification due to its highly compressed index, enabling alignment speeds far exceeding BLAST while preserving alignment precision (Diao et al., 2025). In our simulations, BWA-CMGD achieved high target-species alignment rates and low erroneous assignment rates, while substantially reducing the computational burden relative to BLAST-CMGD. Together, these characteristics support BWA-CMGD as a practical alignment-based strategy for balancing analytical performance and computational efficiency.

Species-level discrimination is clinically important because closely related organisms may differ in pathogenicity and treatment implications, and mis-classification can lead to inappropriate interpretation or antimicrobial selection (Ivens et al., 2025). In our simulations, CMGD-based alignments achieved high target-species alignment rates (>99%) for the tested closely related taxa, including *C. neoformans*/*C. gattii*, *S. aureus*/*S. epidermidis* and *E. coli*/*S. flexneri*. However, high target-species alignment did not necessarily indicate reliable species-level discrimination. This was particularly evident for *E. coli* and *Shigella*, which exhibit profound sequence similarity (Cobo-Simon et al., 2023) and showed extremely low species-discriminatory unique alignment rates (**Figure 5C**). The ambiguity between *E. coli* and *Shigella* is not merely a technical artifact but also reflects an unresolved taxonomic boundary, with previous studies proposing that *Shigella* species should be reclassified as *E. coli* (Parks et al., 2020, 2021). Therefore, resolving highly homologous taxa requires strategies beyond simple unique-read filtering, as species-level assignments based solely on unique reads may be unstable or misleading. Bayesian-based algorithms such as Bracken (Lu et al., 2017) and Pathoscope (Francis et al., 2013) can reassign multi-mapped reads, but their dependence on prior probabilities or unique alignment signals may introduce bias, particularly for low abundance organisms in clinical samples. Complementary approaches, including species-specific databases with conserved regions masked and phylogenetic placement methods, may further improve discrimination among near-neighbor species (Czech et al., 2022). In addition, the spike-in experiment using *S. aureus* and *S. epidermidis* showed that CMGD preserved expected quantitative relationships in mixed samples, despite low-level background cross-alignment. As applicable reporting thresholds for reporting metrics require dedicated validation across different specimen types and sequencing conditions, this study does not define a universal numerical reporting cutoff for species-level clinical reporting. Nevertheless, clinical mNGS results for highly homologous taxa should be interpreted cautiously by integrating genome coverage, discriminatory regions, multi-mapping patterns, clinical context, and orthogonal microbiological evidence. When species-level evidence is insufficient, reporting at the species-complex or genus level may be more appropriate.

Evaluation using public clinical datasets and retrospective internal clinical samples further demonstrated the analytical performance of CMGD in clinical sequencing data. In six published cohorts, BLAST-CMGD showed 91% detection concordance with BLAST-NT and strong correlations in total and uniquely aligned reads, indicating that the curated database retained most of the microbial signals captured by the full NT database. Importantly, most BLAST-NT-exclusive detections were unclassified “sp.” taxa or low-read multi-mapping signals, suggesting that broader database coverage alone does not necessarily yield more interpretable results. In in-house clinical samples, BLAST-CMGD showed strong read-count correlations with BLAST-NT (R² = 0.99 for total reads and R² = 0.89 for uniquely mapped reads). Additional agreement analyses demonstrated high concordance (CCC = 0.99 and 0.92, respectively), while Bland-Altman analysis identified a small positive bias for total mapped reads and a larger positive bias for uniquely mapped reads, indicating a tendency for NT to yield higher read counts, particularly for uniquely mapped reads. Mixed-effects modeling similarly showed no significant overall difference in total mapped reads, whereas uniquely mapped reads were lower with CMGD than with NT. Together, these results indicate that CMGD can reduce database complexity and interpretive ambiguity while preserving most analytical detection signals observed with BLAST-NT.

Despite the robust performance observed in simulated and clinical cohorts, several inherent limitations must be acknowledged. First, CMGD remains primarily dependent on public repositories, including RefSeq and GenBank, and its coverage is therefore constrained by current genome availability, nomenclature accuracy, and database update frequency. The observation that a small subset of pathogens (e.g., unclassified “sp.” taxa) remained detectable only by BLAST-NT suggests that the current database coverage still encounters boundary effects when confronting extremely rare or novel unclassified species (**Figure 6B**). Second, although simulated datasets provide controlled benchmarks, they cannot fully reproduce the complexity of clinical specimens, including nucleic acid degradation, background microbiota, antimicrobial exposure, and contamination. The simulation benchmark was not designed as a strict strain-level hold-out validation, and accession-level non-overlap does not exclude all potential reference-related bias among closely related strains. In addition, the internal clinical evaluation comprised only 30 retrospectively selected mNGS-positive samples. Larger, consecutively enrolled clinical cohorts evaluated against independent microbiological or clinical reference standards will be required to establish diagnostic accuracy. Continuous database updating, periodic re-curation, and multicenter clinical validation will therefore be necessary. Moving forward, annual database updates will remain our core maintenance strategy. By integrating newly generated pathogen data each year, we aim to sustain robust analytical accuracy. For emerging pathogens, the update interval will be shortened to 3–6 months. This strategy enables timely identification of emerging and re-emerging threats, thereby providing a valuable reference for public-health surveillance and response.

In conclusion, this study demonstrates that the analytical performance of mNGS workflows can be improved by jointly optimizing host depletion, microbial database design, and alignment strategy, a principle that underlies the CMGD framework. The combined GRCh38 and T2T host-depletion strategy improves human-read removal while preserving microbial signals, and CMGD provides a clinically focused reference space that reduces ambiguity compared with unfiltered public databases. When coupled with BWA-based alignment, CMGD offers a practical balance among analytical performance, speed, and interpretability. Future work should focus on continuous database updating, systematic removal or flagging of problematic sequences, improved handling of multi-mapped reads, and development of species-complex-aware reporting rules for highly homologous pathogens. These improvements may further strengthen CMGD as a scalable analytical framework for clinical mNGS. Independent prospective and multicenter studies with appropriate clinical reference standards are required to establish its diagnostic accuracy and clinical utility.

## Data Availability

The datasets presented in this study can be found in online repositories. The names of the repository/repositories and accession number(s) can be found in the article/**Supplementary Material**.
