# Supplementary material for: A framework of Microbial Genomic Database for clinical metagenomic pathogen diagnosis: development and multi-cohort evaluation
Source: Front Cell Infect Microbiol. 2026 Sep 16;16:1938149. doi: 10.3389/fcimb.2026.1938149 (PMC13623680; doi:10.3389/fcimb.2026.1938149)
Supplement: Supplementary file 1 [file Table1.docx]

**Supplementary Figures**

**Fig S1. Species identification in residual human reads after host subtraction using BLAST-CMGD, BWA-CMGD, and Kraken2.** (A) Proportion of human reads erroneously and uniquely aligned to microbial sequences. (B) Proportion of human reads erroneously aligned to HHV-6. (C) Proportion of human reads erroneously aligned to non-HHV-6 species. (D) Proportion of endogenous HHV-6 human sequences, annotated as "Homo sapiens isolate … endogenous virus endogenous human herpesvirus 6 sequence", correctly identified.

**Fig S2. Effect of BLAST alignment parameter on results for simulated species.** (A) Alignment rate, unique alignment rate, and erroneous alignment rate for *E. coli* at “max_target_seqs” values of 10, 100, and 500. (B) Corresponding metrics for *K. pneumoniae*.

**Fig S3. Running cpu time of different BLAST alignment parameters.** (A) Different running cpu time for *E. coli* at “max_target_seqs” values of 10, 100, and 500 in simulated datasets within ranged sequence number. (B) Corresponding metrics for *K. pneumoniae*.

**Fig S4. Positive reporting information across public datasets.** (A) Proportion of positive samples in each dataset. (B) Distribution of sample types among positive samples. (C) Top 15 most frequently reported species.

**Fig S5. Alignment details from online NCBI BLAST for four detected samples.** The X-axis and Y-axis represent percent identity and -log10(E-value), respectively. Each point corresponds to a multi-alignment hit for reads mapped to the reported species. Different colors represent distinct sequences, and color intensity indicates point density, with darker shades indicating more overlapping points. Black outlines highlight hits specifically assigned to the reported species. The number of sequences aligned to the literature-reported species is indicated above each plot. For SRR9903834, 16 reads aligned to *S. mitis,* the species reported in the literature, and 15 of these demonstrated identical percent identity and E-values across all multi hits (n=100). The pie charts illustrate the distribution of multi hit species for each read based on 100 hits per read, with hits to *S. mitis* highlighted. Most multiple hits were assigned to *E. faecalis*, *E. faecium*, and *S. aureus*.

**Fig S6. Correlation of detected reads for all species between CMGD and NT in retrospective clinical samples.** (A, B) Species-level total mapped read counts obtained using BLAST-CMGD and BLAST-NT, shown as (A) a correlation scatter plot and (B) a distribution box plot. (C, D) Species-level uniquely mapped read counts obtained using BLAST-CMGD and BLAST-NT, shown as (C) a correlation scatter plot and (D) a distribution box plot.
